# Urinary Frequency as a Presentation of Bulky Malignant Lymphoma in the Pelvis

**DOI:** 10.1155/2013/106820

**Published:** 2013-02-25

**Authors:** Yasukazu Takase, Koichi Kodama, Isamu Motoi, Yasushi Terasaki, Katsuhiko Saito

**Affiliations:** ^1^Department of Urology, Toyama City Hospital, 2-1 Imaizumihokubu-cho, Toyama, Toyama 939-8511, Japan; ^2^Department of Hematology, Toyama City Hospital, 2-1 Imaizumihokubu-cho, Toyama, Toyama 939-8511, Japan; ^3^Department of Pathology, Toyama City Hospital, 2-1 Imaizumihokubu-cho, Toyama, Toyama 939-8511, Japan

## Abstract

Malignant lymphomas may originate from any area of the body and cause a variety of symptoms. However, a malignant lymphoma causing urinary symptoms is uncommon. We report a unique case of a 77-year-old woman who presented with a persistent pollakiuria. Radiographic imaging showed a large pelvic mass (13 × 13 × 11 cm) remarkably compressing and invading the bladder wall and accompanied with bilateral hydronephrosis. Urinary cytology revealed malignant lymphoma, and a final diagnosis of malignant lymphoma was made on the basis of transvaginal needle biopsy. Urinary cytology facilitated the definite diagnosis, following which we initiated a rapid and successful treatment with cyclophosphamide, doxorubicin, vincristine, and prednisone with rituximab.

## 1. Introduction 

Malignant lymphoma (ML) may originate from any area in the body and often causes symptoms such as fever, drenching night sweats, and weight loss. However, the lower urinary tract is an unusual site for early ML manifestations, and patients with ML rarely present with urological symptoms [1]. Herein, we report a case of diffuse large B-cell lymphoma (DLBCL) with a bulky mass (13 × 13 × 11 cm) causing an increased urinary frequency, which was the first evidence of the underlying ML.

## 2. Case Report

**Figure 1:**
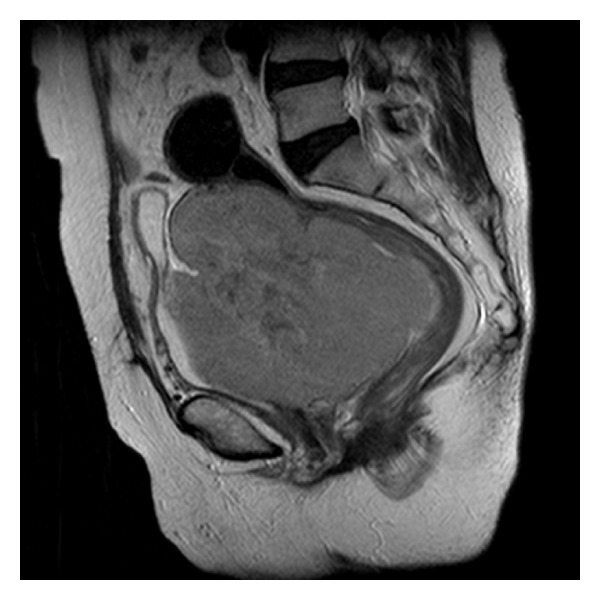
Sagittal T2-weighted magnetic resonance imaging showing a large pelvic mass (13 × 13 × 11 cm) compressing the posterior bladder wall.

**Figure 2:**
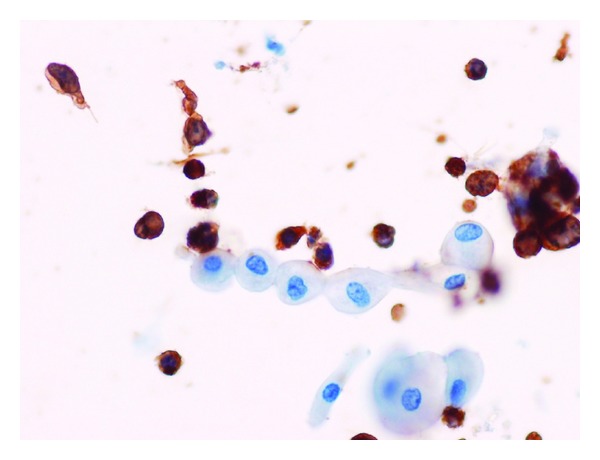
Urinary immunocytology revealing positive staining for leukocyte common antigen.

**Figure 3:**
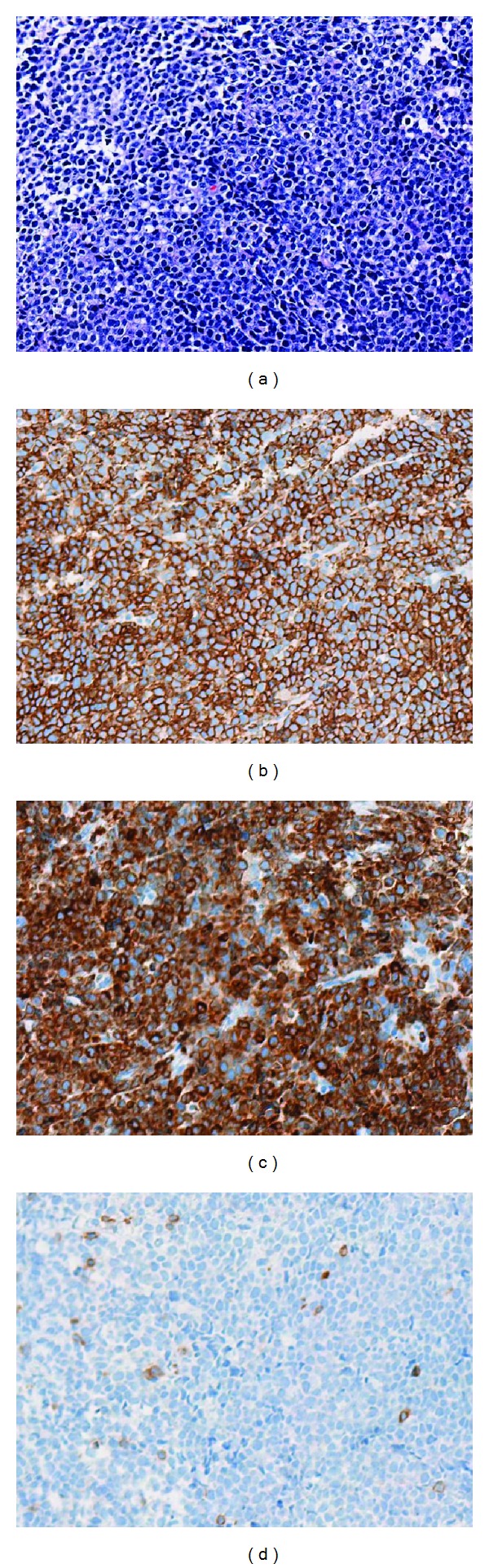
Histopathological examination of a specimen obtained using fine needle biopsy. Lymphoma cells with a high nucleus-to-cytoplasm ratio (hematoxylin and eosin stain) (a). Lymphoma cells showing positive staining for CD20 (b) and CD79a (c) but not CD3 (d).

 A 77-year-old woman was referred to our department, because of increased urinary frequency over the previous month, which had not improved despite the administration of an oral antimuscarinic agent. She lost 3 kg over two months, which was approximately a 5% loss in her body weight. However, she did not report a fever or drenching night sweats. On physical examination, a palpable lower abdominal mass (10 × 15 cm) with elastic consistency, a smooth surface, and mobility, was detected. Palpable lymphadenopathy was not significant. Laboratory data showed slightly elevated levels of serum creatinine (0.97 mg/dL; normal range, 0.4–0.8 mg/dL), C-reactive protein (1.04 mg/dL; normal range, 0.00–0.30 mg/dL), and lactate dehydrogenase (885 IU/l; normal range, 110–210 IU/L). Urinalysis results were close to normal. Abdominal computed tomography (CT) revealed a lobulated mass (13 × 13 × 11 cm) in the pelvis, adjacent to the left pelvic wall. The mass compressed the urinary bladder anteriorly, possibly invaded the posterior bladder wall, and resulted in bilateral hydronephrosis. No apparent lymphadenopathy or metastasis was revealed. Magnetic resonance imaging (MRI) showed a tumor demonstrating hypointensity on T1-weighted and hyperintensity on T2-weighted imaging. MRI also demonstrated the tumor invasion of the bladder wall (Figure 1). These findings led to the suspicion of a sarcoma originating in the pelvic space. However, urinary cytology revealed a few scattered atypical cells, which were pleomorphic and had high nucleus-to-cytoplasm ratios. Furthermore, urinary immunocytology revealed that these atypical cells showed positive immunohistochemical staining for leukocyte common antigen, strongly indicating ML (Figure 2). Ultrasonography-guided transvaginal fine needle biopsy of the mass was performed. Histopathologically, the specimens consisted of diffuse, proliferative, small round cells with a high nucleus-to-cytoplasm ratio, which were positive for CD20, CD79a, and Bcl2 but negative for CD3 (Figure 3). Besides the pelvic mass, positron emission tomography (PET)/CT showed no abnormal accumulations in any other tissues. A final diagnosis of DLBCL stage IIEA was made on the basis of the Ann Arbor classification. After six courses of cyclophosphamide, doxorubicin, vincristine, and prednisone with rituximab (R-CHOP), the level of soluble interleukin-2 receptor decreased from 39200 to 297 U/mL (normal range, 145–519 U/mL) and PET/CT showed no abnormal uptake. Urinary cytology revealed no abnormal cells. Complete remission was achieved and has lasted for one year to date.

## 3. Discussion

Clinically, urogenital secondary involvement of ML is less frequent, especially involvement of the bladder. Watson et al. described the following classification for secondary vesical lymphoma: (1) circumscribed single or multiple foci limited to the bladder wall, (2) direct invasion of the bladder by a perivesical tumor, and (3) vesical extension from prostatic foci [2]. In a review of autopsy or antemortem data of 1068 patients with ML, urogenital involvement was detected in 72 patients (6.7%) and vesical involvement was detected in only two patients (0.2%) [3].

Urinary frequency as a symptom of secondary ML of the bladder is uncommon, excluding gross bladder involvement. In 72 patients with microscopic vesical infiltration, 10 patients reported vesical symptoms during their lifetime, which occurred relatively late in the course of the disease [4]. In the present case, a bulky mass compressed the patient's bladder and decreased its capacity, which may have resulted in the early symptom of increased urinary frequency.

Urinary cytology is a diagnostic method to detect urinary tract malignancies and may be useful to diagnose primary or secondary ML of the urinary tract. In a report of 50 consecutive patients with ML, 14 (28%) had positive cytological findings [5]. However, lymphoma cells are very fragile in urine sediments [6], and it is difficult to obtain sufficient material for immunocytological staining [7]. 

It remains controversial whether a bulky disease influences the efficacy of R-CHOP, a standard treatment for DLBCL. In some reports, bulky diseases were not prognostic factors in subgroup analyses [8, 9]. In contrast, the maximum tumor diameter is an important prognostic factor for progression-free survival and overall survival in DLBCL patients receiving R-CHOP [10, 11]. However, further studies are needed to determine whether a bulky mass is also a prognostic factor for DLBCL.
